# Development of an Orchard Inspection Robot: A ROS-Based LiDAR-SLAM System with Hybrid A*-DWA Navigation

**DOI:** 10.3390/s25216662

**Published:** 2025-11-01

**Authors:** Jiwei Qu, Yanqiu Gu, Zhinuo Qiu, Kangquan Guo, Qingzhen Zhu

**Affiliations:** 1 School of Mechanical Engineering, Yangzhou University, Yangzhou 225127, China; mz120250922@stu.yzu.edu.cn (Y.G.);; 2 College of Mechanical and Electronic Engineering, Northwest A&F University, Xianyang 712100, China; 3 School of Agricultural Engineering, Jiangsu University, Zhenjiang 212013, China

**Keywords:** intelligent equipment, mobile robot, SLAM, autonomous navigation, obstacle avoidance, information perception

## Abstract

The application of orchard inspection robots has become increasingly widespread. How-ever, achieving autonomous navigation in unstructured environments continues to pre-sent significant challenges. This study investigates the Simultaneous Localization and Mapping (SLAM) navigation system of an orchard inspection robot and evaluates its performance using Light Detection and Ranging (LiDAR) technology. A mobile robot that integrates tightly coupled multi-sensors is developed and implemented. The integration of LiDAR and Inertial Measurement Units (IMUs) enables the perception of environmental information. Moreover, the robot’s kinematic model is established, and coordinate transformations are performed based on the Unified Robotics Description Format (URDF). The URDF facilitates the visualization of robot features within the Robot Operating System (ROS). ROS navigation nodes are configured for path planning, where an improved A* algorithm, combined with the Dynamic Window Approach (DWA), is introduced to achieve efficient global and local path planning. The comparison of the simulation results with classical algorithms demonstrated the implemented algorithm exhibits superior search efficiency and smoothness. The robot’s navigation performance is rigorously tested, focusing on navigation accuracy and obstacle avoidance capability. Results demonstrated that, during temporary stops at waypoints, the robot exhibits an average lateral deviation of 0.163 m and a longitudinal deviation of 0.282 m from the target point. The average braking time and startup time of the robot at the four waypoints are 0.46 s and 0.64 s, respectively. In obstacle avoidance tests, optimal performance is observed with an expansion radius of 0.4 m across various obstacle sizes. The proposed combined method achieves efficient and stable global and local path planning, serving as a reference for future applications of mobile inspection robots in autonomous navigation.

## 1. Introduction

The automation of agricultural monitoring is crucial for enhancing orchard management efficiency and reducing labor intensity [1]. Among various technological solutions, autonomous mobile robots have emerged as a promising platform due to their capability for flexible, real-time, and continuous inspection [2,3]. Compared to alternative methods such as drone surveillance [4], and fixed-point monitoring [5], mobile robots offer a more balanced approach for detailed, ground-level data collection.

However, realizing effective autonomous navigation and obstacle avoidance of mobile robots in unstructured orchard environments poses significant challenges. Developing an appropriate autonomous navigation method remains a key area of research in the development of mobile robots [6,7]. Under shaded conditions, Global Navigation Satellite System (GNSS) signals are often unreliable, prompting the use of vision-based navigation and Light Detection and Ranging (LiDAR) Simultaneous Localization and Mapping (SLAM) as primary alternatives [8,9,10]. LiDAR SLAM is frequently preferred due to its high accuracy, adaptability to varying lighting conditions, robust real-time performance, and ability to navigate complex environments [11,12].

Currently, LiDAR SLAM for mobile robots is primarily developed based on the Robot Operating System (ROS) technology. ROS provides modular architecture for mobile robots, supports multi-language and cross-platform development, and features a rich toolchain, such as Rviz and Gazebo, along with standardized communication mechanisms compatible with various hardware platforms [13,14]. In ROS-based mobile robot navigation technologies, such as map construction and localization, path planning, and motion control [15,16,17], addressing issues related to the configuration of ROS coordinate frames is essential. Errors in the orientation of the LiDAR transform (TF) coordinate frame can result in mismatches between point clouds and map orientation, while TF conflicts may result in model jitter. Additionally, the loss of LiDAR data can cause failures in the Adaptive Monte Carlo Localization (AMCL), making it necessary to conduct a thorough inspection of the TF tree’s integrity and to incorporate appropriate coordinate transformations. Moreover, if the parameters of the local planner do not match environmental conditions, adjustments to the expansion radius and obstacle distance will be required. Therefore, the development of a robust kinematic model for robots, and precise configuration of navigation parameters are essential for efficient path planning of mobile robot.

Path planning algorithms are also key factors in achieving autonomous navigation for mobile robots [18]. These methods can be categorized into global path planning and local path planning. Commonly used global path planning algorithms include Dijkstra, Rapidly-exploring Random Tree (RRT), ant colony optimization, and A* methods [19]. Research reveals A*’s superiority in processing efficiency, travel duration, distance optimization, and energy consumption when benchmarked against breadth-first and depth-first search approaches [20]. Local planners, such as the Dynamic Window Approach (DWA), excel in real-time obstacle avoidance by evaluating kinematically feasible velocities [21,22]. To harness the strengths of both, hybrid navigation strategies have received extensive research and attention. The Lightweight and Ground-Optimized LiDAR Odometry and Mapping on variable terrain (LeGO-LOAM) method demonstrated excellent performance in RRT* path planning [23]. A novel full-coverage approach in hybrid navigation achieved a coverage rate of 98% [24]. Moreover, an integrated framework combining optimized A* with DWA has demonstrated enhanced operational intelligence and navigation efficiency through experimental validation [25]. Furthermore, a refined methodology that synergizes enhanced A* with adaptive DWA has achieved smoother trajectories and an 18.6% improvement in search efficiency in complex scenarios [26]. This combined algorithm significantly improved the efficiency and reliability of agricultural robot mobility. While current methods have shown promising results in mobile robot navigation, the real-world validations of such hybrid strategies for inspection robots in complex, unstructured orchards remain limited. To address these limitations and realize reliable inspections, this study proposes the development and experimental validation of a ROS-based LiDAR-SLAM system for an orchard inspection robot, employing a hybrid A*-DWA path planning strategy. The hypothesis is that a tightly coupled LiDAR- Inertial Measurement Unit (IMU) SLAM system combined with an adaptive A*-DWA path planner will achieve more accurate and robust autonomous navigation in unstructured orchard environments compared to conventional methods. The experimental validation successfully demonstrates improved path accuracy and obstacle avoidance performance.

The main contributions of this study are defined as follows: a SLAM navigation system for inspection robots was developed based on the construction of a robotic kinematic model and the design of ROS navigation function modules. A path planning method combining the A* and DWA algorithms was designed for global and local path planning. The robot’s path navigation accuracy and obstacle avoidance performance parameters were calculated through outdoor experiments in unstructured environments.

Finally, the remaining of the paper is organized as follows: Section 2 outlines the materials and methods, including system components, path planning strategies, and experimental procedures. Section 3 presents the results’ analysis, while Section 4 and Section 5 are devoted to the discussion, conclusions and future works, respectively.

## 2. Materials and Methods

### 2.1. Robot System

**Figure 1:**
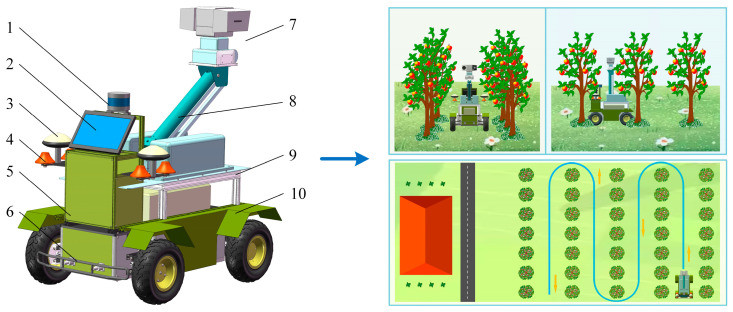
Structure of the mobile inspection robot and schematic diagram of inspection operations. 1. LiDAR; 2. Tablet computer; 3. GNSS antenna; 4. Communication antenna; 5. Control box; 6. Collision-prevention pole; 7. Camera; 8. Elevating pole; 9. Cargo platform; 10. Mudguard.

The structure of the mobile inspection robot and the schematic diagram of inspection operations are illustrated in Figure 1. The robot is composed of three main components: a four-wheel independently driven chassis, a navigation system, and a camera platform. The navigation system includes an industrial computer, an IMU, a LiDAR system, a power supply system, a gimbal camera, a GNSS module, and communication lines. The industrial computer (NP-6122-JH3, Nodajia Industrial Control Co., Ltd., Shanghai, China) processes sensor data and implements path planning algorithms, successively transmitting motion control commands to the chassis. Moreover, the IMU (WT901C-9, Victory Intelligence Technology Co., Ltd., Beijing, China) measures the robot’s speed, acceleration, attitude angle, and other motion parameters to provide accurate pose information. As for the LiDAR system (RS-LiDAR-16, RoboSense Technology Co., Ltd., Shenzhen, China), it collects environmental data surrounding the robot, which is essential for map construction.

The robot is built as a four-wheel drive differential-steering mobile platform, known for its strong driving force and flexible steering. To ensure optimal movements and shock absorption, the robot chassis incorporates a double-arm independent suspension mechanism that minimizes impact on the actuators. For inspection tasks, a lifting rod combined with a gimbal camera is employed, allowing for image capture of different crops at varying heights and angles.

### 2.2. Kinematic Model of Robot

#### 2.2.1. Transformation of the Robot Coordinate System

**Figure 2:**
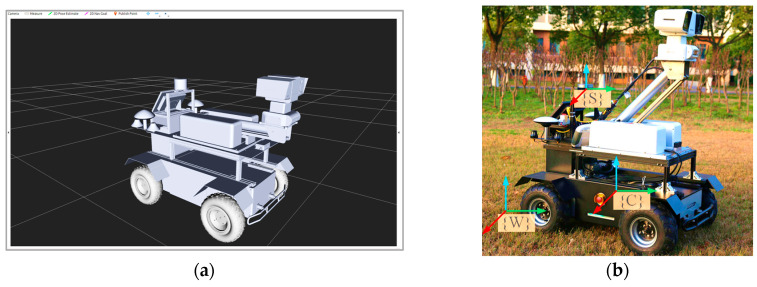
Schematic diagram of the URDF model (**a**) and the robot coordinate system (**b**).

The Unified Robotics Description Format (URDF) is an Extensible Markup Language (XML) used for describing robots. In the ROS environment, URDF enables the visualization of robot features, such as shape and profile, through the Rviz tool. Given the complex structure of the robot considered in this paper, constructing the robot model solely using XML statements presents significant challenges. To address this issue, SolidWorks 2022 software was used as it provides model conversion capabilities. Thus, the *sw_urdf_exporter* plugin was employed to convert the designed 3D model into a URDF file compatible with ROS, as illustrated in Figure 2a.

The transformation between two Cartesian coordinate systems follows the Helmert seven-parameter transformation model. Let the coordinates of the same point in the two coordinate systems be represented as (*x*_1_, *y*_1_, *z*_1_) and (*x*_2_, *y*_2_, *z*_2_), respectively. Consequently, the following relationship can be established:

(1) $$\left[\begin{array}{c} x_{1}\\ y_{1}\\ z_{1} \end{array}\right]=\left[\begin{array}{c} \Delta x\\ \Delta y\\ \Delta z \end{array}\right]+k\cdot R_{x}\left(\delta \right)\cdot R_{y}\left(\varphi \right)\cdot R_{z}\left(\theta \right)\left[\begin{array}{c} x_{2}\\ y_{2}\\ z_{2} \end{array}\right]$$

where Δ*x*, Δ*y*, and Δ*z* denote the displacements of a point along the three axes in one of the coordinate systems. $R_{x}\left(\delta \right)$, $R_{y}\left(\varphi \right)$, and $R_{z}\left(\theta \right)$ represent the rotation amounts around the respective axes, and *k* indicates the scale factor between the two coordinate systems. *δ*, *φ*, and *θ* are Euler angles, specifying the rotation angles around the three axes.

Furthermore, matrices $R_{x}\left(\delta \right)$, $R_{y}\left(\varphi \right)$, and $R_{z}\left(\theta \right)$ are expressed as follows:

(2) $$R_{x}\left(\delta \right)=\left[\begin{array}{ccc} 1 & 0 & 0\\ 0 & \cos\delta & -\sin\delta \\ 0 & \sin\delta & \cos\delta \end{array}\right]$$

(3) $$R_{y}\left(\varphi \right)=\left[\begin{array}{ccc} \cos\varphi & 0 & \sin\varphi \\ 0 & 1 & 0\\ -\sin\varphi & 0 & \cos\varphi \end{array}\right]$$

(4) $$R_{z}\left(\theta \right)=\left[\begin{array}{ccc} \cos\theta & -\sin\theta & 0\\ \sin\theta & \cos\theta & 0\\ 0 & 0 & 1 \end{array}\right]$$

During robot operation, sensors, such as the LiDAR, IMU, and GNSS, operate within their own independent coordinate systems. To facilitate data fusion, the definition of the relative positional relationships among these sensors is required. Consequently, we define the world coordinate system (*W*), the vehicle coordinate system (*C*), and the LiDAR coordinate system (*S*), as illustrated in Figure 2b.

LiDAR point cloud data is crucial for the robot’s environmental perception, significantly aiding in the acquisition of the vehicle’s position and obstacle information. Therefore, we illustrate the coordinate transformation process with respect to LiDAR. Assume that the coordinates of any point in the environment are represented as (*x_s_*, *y_s_*, *z_s_*) in the LiDAR coordinate system (*S*), and as (*x_c_*, *y_c_*, *z_c_*) in the vehicle coordinate system (*C*). The LiDAR’s displacement along the three axes of the robot’s body coordinate system (*C*) is denoted as Δ*x*, Δ*y*, and Δ*z*, collectively labeled as (*x_t_*, *y_t_*, and *z_t_*). Furthermore, the LiDAR undergoes rotations of *δ*, *φ*, and *θ* radians around the *x*, *y*, and *z* axes, respectively. It is important to note that there is no scale change between the vehicle coordinate system (*C*) and the LiDAR coordinate system (*S*); hence, we scale factor *k* is set to one. Based on Equation (1), the following coordinate transformation relationship can be derived:

(5) $$\left[\begin{array}{c} x_{c}\\ y_{c}\\ z_{c} \end{array}\right]=\left[\begin{array}{c} x_{t}\\ y_{t}\\ z_{t} \end{array}\right]+R_{x}\left(\delta \right)\cdot R_{y}\left(\varphi \right)\cdot R_{z}\left(\theta \right)\left[\begin{array}{c} x_{s}\\ y_{s}\\ z_{s} \end{array}\right]$$

By substituting the rotation matrices $R_{x}\left(\delta \right)$, $R_{y}\left(\varphi \right)$, and $R_{z}\left(\theta \right)$ into Equation (5), we can deduce Equation (6), as follows:

(6) $$\left[\begin{array}{c} x_{c}\\ y_{c}\\ z_{c} \end{array}\right]=\left[\begin{array}{c} x_{t}\\ y_{t}\\ z_{t} \end{array}\right]+\left[\begin{array}{ccc} 1 & 0 & 0\\ 0 & \cos\delta & -\sin\delta \\ 0 & \sin\delta & \cos\delta \end{array}\right]\left[\begin{array}{ccc} \cos\varphi & 0 & \sin\varphi \\ 0 & 1 & 0\\ -\sin\varphi & 0 & \cos\varphi \end{array}\right]\left[\begin{array}{ccc} \cos\theta & -\sin\theta & 0\\ \sin\theta & \cos\theta & 0\\ 0 & 0 & 1 \end{array}\right]\left[\begin{array}{c} x_{s}\\ y_{s}\\ z_{s} \end{array}\right]$$

Utilizing Equation (6), any point can be transformed between the LiDAR coordinate system (*S*) and the vehicle coordinate system (*C*). When integrated with the ROS coordinate transformation tool, this allows us to establish the TF coordinate relationships for the robot.

#### 2.2.2. Forward Kinematics

**Figure 3:**
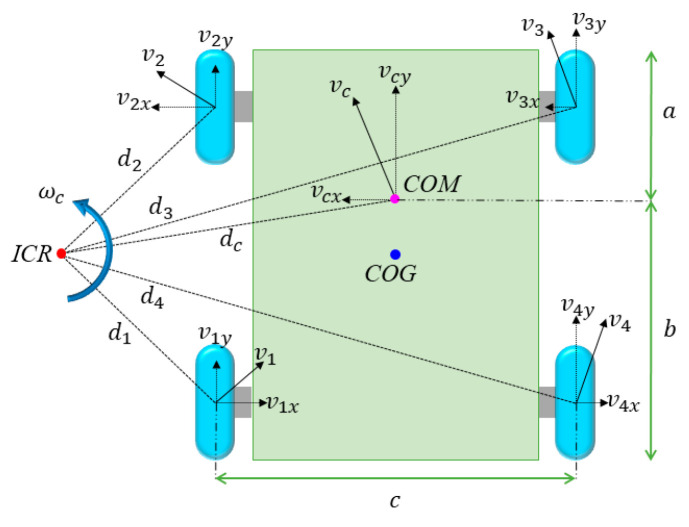
Schematic diagram of the kinematic model for the robot chassis.

The kinematic model of the four-wheel differential drive mobile robot is illustrated in Figure 3. The geometric center of the chassis is located at point *COG*, whereas the center of rotation during the chassis steering motion is at point *ICR*, with points *ICR* and *COG* being co-axial. The distance from point *ICR* to each wheel is denotes as *d_i_*, while *v_i_* represents the actual velocity of each wheel, which can be decomposed into two components: the lateral sliding velocity *v_ix_* and the target velocity *v_iy_*, where *i* = 1, 2, 3, and 4. When the chassis slides laterally, the instantaneous velocity center is located at the center of mass, denoted as *COM*. The velocity of the chassis can be represented by the linear velocity *v_c_* and the angular velocity *ω_c_* at this point. The distance between point *COM* and point *ICR* is *d_c_*, where *v_c_* is perpendicular to the line connecting points *COM* and *ICR*. The velocity *v_c_* can be further decomposed into lateral sliding velocity *v_cx_* and target velocity *v_cy_*. The distance between the centerlines of the wheels on the left and right sides of the chassis is denoted as *c*, while the distances from point *COM* to the front and rear ends of the chassis are represented by *a* and *b*, respectively.

The relationship between the linear velocity *v_c_*, angular velocity *ω_c_*, and the radius of motion *d_c_* is expressed in Equation (7).

(7) $$\omega_{c}=\frac{v_{c}}{d_{c}}$$

During the rotation of a rigid body, the angular velocity at any point within the body is equivalent to the angular velocity at the *COM*. We can derive the following relationship:

(8) $$\omega_{c}=\frac{v_{\mathrm{ix}}}{d_{\mathrm{iy}}}=\frac{v_{\mathrm{iy}}}{d_{\mathrm{ix}}}$$

The projections of *d_i_* and *d_c_* onto the *x*-axes and *y*-axes have lengths that satisfy Equations (9) and (10), respectively.

(9) $$d_{1x}=d_{2x}=d_{\mathrm{cx}}-\frac{c}{2}$$

(10) $$d_{3x}=d_{4x}=d_{\mathrm{cx}}+\frac{c}{2}$$

Let the speeds of the left and right wheels of the four-wheel differential chassis be denoted as *V_L_* and *V_R_*, respectively. When the speeds of the front and rear wheels are synchronized, the relationships given in Equations (11) and (12) can be established.

(11) $$V_{L}=v_{1y}=v_{2y}$$

(12) $$V_{R}=v_{3y}=v_{4y}$$

The relationships established in Equations (6)–(12) allow to derive Equations (13) and (14).

(13) $$V_{L}=\omega_{c}\cdot \left(d_{\mathrm{cx}}-\frac{c}{2}\right)=\omega_{c}d_{\mathrm{cx}}{-\omega }_{c}\frac{c}{2}=v_{\mathrm{cy}}-\omega_{c}\frac{c}{2}$$

(14) $$V_{R}=\omega_{c}\cdot \left(d_{\mathrm{cx}}+\frac{c}{2}\right)=\omega_{c}d_{\mathrm{cx}}+\omega_{c}\frac{c}{2}=v_{\mathrm{cy}}+\omega_{c}\frac{c}{2}$$

Concerning Equations (13) and (14), they yield the forward kinematic relationships of the four-wheel differential chassis, as expressed in Equation (15).

(15) $$\left[\begin{array}{c} v_{\mathrm{cy}}\\ \omega_{c} \end{array}\right]=\left[\begin{array}{cc} \frac{1}{2} & \frac{1}{2}\\ -\frac{1}{c} & \frac{1}{c} \end{array}\right]\left[\begin{array}{c} V_{L}\\ V_{R} \end{array}\right]$$

#### 2.2.3. Inverse Kinematics

The process of calculating the overall speed of the chassis based on the speeds of individual wheels is referred to as forward kinematics. Conversely, inverse kinematics consists of defining the speeds of each wheel from the overall speed. By applying matrix inversion to Equations (15) and (16) can be derived.

(16) $$\left[\begin{array}{c} V_{L}\\ V_{R} \end{array}\right]=\left[\begin{array}{cc} 1 & -\frac{c}{2}\\ 1 & \frac{c}{2} \end{array}\right]\left[\begin{array}{c} v_{\mathrm{cy}}\\ \omega_{c} \end{array}\right]$$

### 2.3. Path Planning Configuration

**Figure 4:**
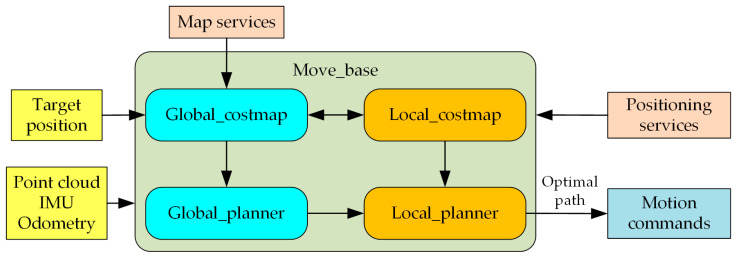
Framework for the robot autonomous navigation system program.

This robot operates on Ubuntu 18.04 as its software platform within the Linux environment and utilizes the ROS as its runtime framework. In ROS, several processes communicate between nodes, functioning within a distributed architecture. This design enables the robot’s nodes to receive, publish, and reuse messages related to sensor data, packages, libraries, and other information via point-to-point communication. Within the ROS computational graph, the Master maintains the registration information for the topics and services associated with nodes. When nodes communicate with the Master, they can establish mutual connections, allowing the Master to provide feedback that ensures precise data updates. The programming languages used for the development of this robot are C++ and Python, with Visual Studio Code (Version 1.95.3, Redmond, WA, USA) serving as the development tool. ROS provides developers with a comprehensive software framework known as *move_base*. The navigation control system for the robot discussed in this work is designed based on this framework, as illustrated in Figure 4. The *move_base* navigation package in ROS offers essential runtime nodes and interfaces for robot navigation, with algorithm implementation code integrated into the framework as plugins. Initially, *move_base* acquires point cloud information by subscribing to messages in the *sensor_msgs/LaserScan* and *sensor_msgs/PointCloud* formats, published by the LiDAR runtime nodes. Secondly, it subscribes to odometry data in the *nav_msgs/Odometry* format and to transformation data via *tf* to perform motion calculations. Finally, it publishes command topics in the *geometry_msgs/Twist* format to regulate the robot’s linear and angular velocities, thereby facilitating effective motion control.

(1) Configuration of the Costmap Plugin

Within the framework of the robot’s autonomous navigation system, two types of costmaps are employed to represent obstacle information in the environment: the first for global path planning (*global_costmap*), whereas the second is for local path planning and obstacle avoidance (*local_costmap*). The configuration of these costmaps encompasses general configuration, global planning configuration, and local planning configuration.

(i) General Plugin Configuration

The specific parameters for the general costmap configuration are defined as follows:

- *footprint* defines the area occupied by the robot on the map;
- *obstacle_range* specifies the maximum detection range for obstacles, set to 2.5 m;
- *raytrace_range* denotes the maximum range for spatial detection, set to 3.0 m;
- *min_obstacle_height* represents the minimum height of obstacles, set to 0.25 m;
- *max_obstacle_height* denotes the maximum height of obstacles, set to 0.35 m;
- *observation_sources* declares the required sensor information for the map and is set to *scan*, which refers to LiDAR point cloud data;
- *inflation_radius* sets the expansion radius around obstacles.

(ii) Global Costmap Plugin Configuration

The parameters for the global costmap plugin configuration include the following:

- *global_frame* identifies the reference frame for the global costmap. It is set to *map*, corresponding to the world coordinate system;
- *robot_base_frame* specifies the robot’s coordinate frame used as a reference for the costmap. It is set to *base_link*, which refers to the robot’s coordinate system discussed in this paper;
- *update_frequency* indicates the frequency at which map information is updated and is set to 1 Hz.

(iii) Local Costmap Plugin Configuration

The parameters for the local costmap plugin configuration are defined as follows:

- *global_frame*, *robot_base_frame*, and *update_frequency* align with those used in the global planning configuration;
- *publish_frequency* specifies the frequency at which the map information is visualized, set to 2 Hz;
- *width*, *height*, and *resolution* define the dimensions and resolution of the costmap, set to 10 m, 10 m, and 0.5 m, respectively.
(2) Configuration of the Path Planning Plugin

The primary function of command planning is to compute path information and convert it into speed control commands for the robot. This configuration shows that the robot utilizes A* algorithm for global planning and DWA algorithm for local planning.

(3) Launching the Navigation Nodes

Following the configurations detailed in Sections (1) and (2), the setup of the relevant algorithm plugins within the autonomous navigation system framework for the mobile robot has been completed. Additionally, a launch file is required to load parameters from the configuration file. This launch file is entitled *robuster_mr_navigation.launch*. By utilizing this launch file, the necessary nodes and plugins for operating the navigation system can be activated, enabling the conversion of path information into speed control commands, and thus facilitating the movement of the robot’s chassis.

### 2.4. Path Planning Scheme

Based on the analysis of commonly used path planning algorithms discussed previously, this paper proposes a combined approach utilizing an A* algorithm and DWA for the path planning of agricultural robots.

The cost function *f*(*n*) of the A* algorithm is expressed as Equation (17):

(17) f(n)=g(n)+h(n)

where *n* denotes the current node; *g*(*n*) represents the actual distance cost from the start node *s* to *n*; and *h*(*n*) denotes the estimated distance cost from *n* to the goal node *g*. The heuristic *h*(*n*) controls the search direction and node expansion order in A*, playing a critical role in ensuring the algorithm’s optimality.

In the A* algorithm, the distance metric is typically represented by the Euclidean distance. Accordingly, this study employs the Euclidean distance to compute both *g*(*n*) and *h*(*n*), as defined in Equations (18) and (19), respectively.

(18) $$g\left(n\right)=\sqrt{{\left(x_{n}-x_{s}\right)}^{2}+{\left(y_{n}-y_{s}\right)}^{2}}$$

(19) $$h\left(n\right)=\sqrt{{\left(x_{g}-x_{n}\right)}^{2}+{\left(y_{g}-y_{n}\right)}^{2}}$$

where (*x_s_*, *y_s_*), (*x_n_*, *y_n_*), and (*x_g_*, *y_g_*) denote the coordinates of the start node, current node, and goal node in the grid map, respectively.

In the conventional A* algorithm, the heuristic function assigns equal weights (i.e., unity weighting) to both the heuristic cost *h*(*n*) and the actual cost *g*(*n*). This approach often results in the expansion of an excessive number of candidate nodes, thereby diminishing search efficiency. An effective adaptive weight function should emphasize exploration in the early stages and exploitation in later phases. To achieve this, it is advisable to begin with a small weight that promotes broad exploration, gradually increasing it as the search accumulates cost information. This approach concentrates the search on promising areas, thereby accelerating convergence. Additionally, ensuring a smooth transition is essential to maintain stability.

Guided by this principle, the specific weight function in Equation (20), was formulated. The normalized progress parameter $\frac{d}{D}$ naturally serves as the independent variable. The bounds of $\frac{d}{D}$ is from 0.5 to 1.5, which were determined empirically through simulation across a variety of test scenarios. A weight lower than 0.5 led to excessive, Dijkstra-like exploration, while a weight consistently above 1.5 risked behaving like a greedy best-first search, compromising path optimality. To bound the weight within the effective interval [0.5, 1.5], a sinusoidal function was selected for its inherent smoothness and non-linear properties. It initializes the weight near 0.5 when $\frac{d}{D}$ is small to foster exploration, and drives it towards 1.5 as $\frac{d}{D}$ approaches 1 to expedite convergence. The specific bounds were empirically validated to prevent the algorithm from degenerating into a brute-force search (if weights are too low) or a shortsighted greedy search (if weights are too high), thus achieving a robust balance across various environments.

(20) $$f\left(n\right)=g\left(n\right)+\{\sin^{2}[\frac{\pi }{2}(1-\frac{d}{D})]+\frac{1}{2}\}h(n)$$

where *d* represents the distance from the start node to the current node, and *D* is the total distance from the start node to the goal node.

**Figure 5:**
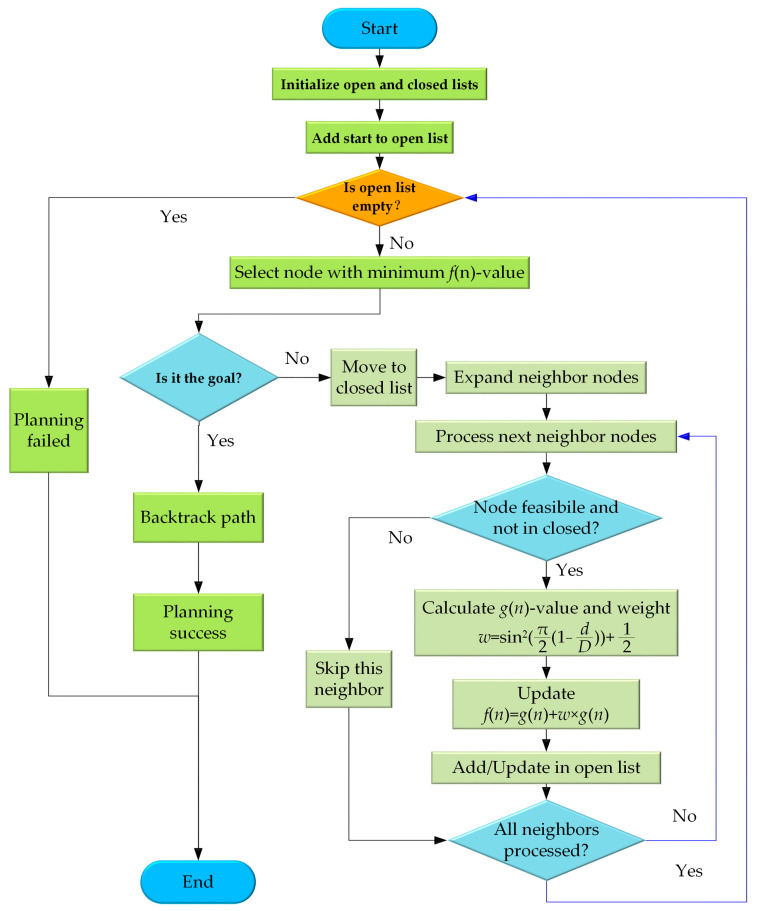
Flowchart of the improved A* algorithm.

This flowchart for the improved A* path planning algorithm is illustrated in Figure 5. The process initializes open and closed lists, adding the start node to the open list. The core loop continues while nodes remain in the open list. Each iteration selects the node with the minimum *f*(*n*)-value. If this node is the goal, the path is reconstructed, marking success. Otherwise, it moves to the closed list, and its neighbors are expanded. For each feasible neighbor not in the closed list, the algorithm calculates the actual cost (*g*(*n*)-value) and a key adaptive weight (*w*). This weight dynamically scales the heuristic cost (*h*(*n*)-value), updating the node’s *f*(*n*)-value. The node is then added or updated in the open list. This process repeats for all neighbors before the loop continues, ensuring efficient exploration. Exhausting the open list without finding the goal results in a failure state, terminating the process.

The DWA algorithm assesses multiple motion trajectories that comply with constraint conditions using an evaluation function that encompasses three dimensions. It prioritizes trajectories based on three criteria: (1) minimizing the angle between the trajectory endpoint and the target point, (2) maximizing the distance from obstacles, and (3) optimizing for higher velocities. The formulation of the evaluation function is as follows:

(21) $$G\left(v,\;\omega \right)=\sigma \left[\alpha \cdot \mathrm{head}\left(v,\;\omega \right)+\beta \cdot \mathrm{dist}\left(v,\;\omega \right)+\gamma \cdot \mathrm{vel}\left(v,\;\omega \right)\right]$$

where *σ* is the normalization coefficient, while *α*, *β*, and *γ* are the weight coefficients. *head*(*v*, *ω*) refers to the angular deviation between the orientation of the trajectory endpoint and the target point, and smaller deviations yield higher evaluations. *dist*(*v*, *ω*) reflects the minimum distance from the trajectory endpoint to the nearest obstacle, with larger values indicating a greater distance from obstacles. *vel*(*v*, *ω*) represents the robot’s velocity, where higher speeds correspond to more favorable evaluations.

The weight coefficients (*α*, *β*, *γ*) in the DWA evaluation function (Equation (21)) were empirically determined and tuned to prioritize safe and goal-directed navigation in unstructured orchard environments. Through extensive simulation and subsequent field testing, the final parameter set was configured as *α* = 0.6, *β* = 0.3, and *γ* = 0.1. This configuration assigns the highest priority to the goal-directed term (*α*) to ensure proactive movement towards target waypoints, while maintaining a significant weight on obstacle clearance (*β*) for safety. The velocity term (*γ*) was assigned a relatively lower weight to prevent aggressive motions that could lead to instability on uneven terrain. This specific weighting scheme was found to provide the best balance, enabling the robot to navigate efficiently while robustly avoiding collisions in the complex test environment.

**Figure 6:**
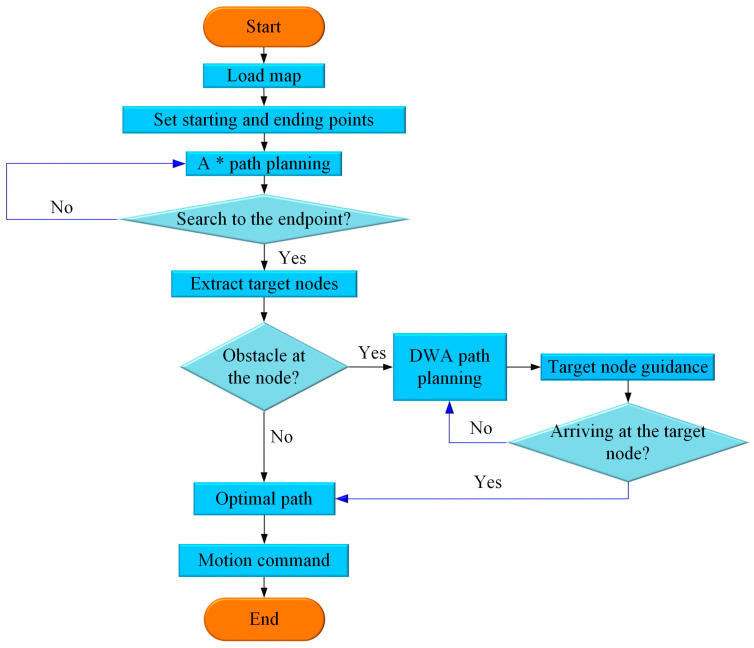
Flowchart of path planning based on the hybrid A*-DWA strategy.

This integrated path planning scheme aims to enhance the efficiency of field information collection robots. The proposed methodology involves the following steps: initially, the nodes traversed by the global path defined by the A* algorithm act as guiding nodes. When obstacles are encountered along this global path, these guiding nodes serve as target nodes in local path planning. As a result, the optimal path is determined, and motion commands are issued to the robot to facilitate obstacle avoidance. The flowchart of this process is illustrated in Figure 6.

### 2.5. Simulation and Experiment of Motion Performance

#### 2.5.1. Simulation for Path Planning Approach

**Figure 7:**
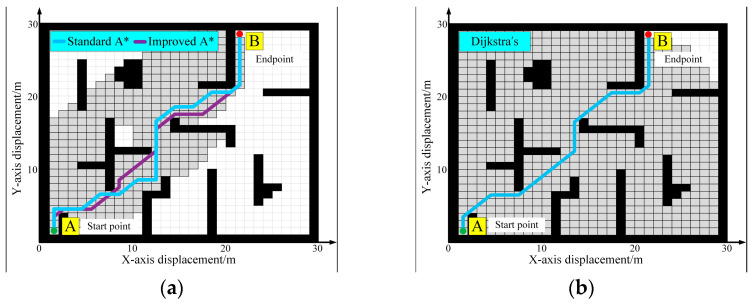
Simulation of global path planning algorithms: (**a**) A* algorithm; (**b**) Dijkstra’s algorithm.

To validate the effectiveness of the proposed path planning approach, simulations were conducted using Matlab R2022b through 2D grid. While 2D grid simulations do not fully replicate real-world robotic environments, they nevertheless provide a comprehensive means for evaluating critical aspects of path planning algorithms—such as search efficiency, path length, and smoothness. Thus, these simulations serve as a well-suited approach for the initial assessment of algorithmic performance. For global path planning performance, the simulation results of the standard A* algorithm and the improved A* algorithm are shown in Figure 7a. In the standard A* algorithm, the path planning time is 10.5 s, the path length is 44.32 m, and a total of 427 nodes are traversed. In the improved A* algorithm, the time and length for path planning are 4.3 s and 38.79 m, respectively, and a total of 374 nodes are traversed. Additionally, we also compared the improved A* algorithm to another alternative method, specifically Dijkstra’s algorithm. The result of Dijkstra’s algorithm is illustrated in Figure 7b. The time and length for path planning are 18.7 s and 38.79 m, respectively, and a total of 684 nodes are traversed. The improved A* algorithm proves to be more efficient, requiring less time and exploring fewer nodes compared to the standard A* algorithm and Dijkstra’s algorithm.

**Figure 8:**
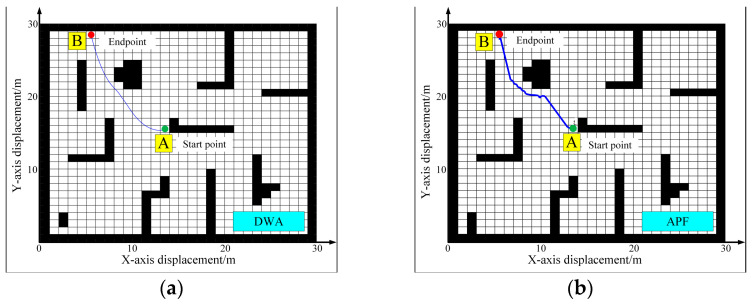
Simulation of local path planning algorithms: (**a**) DWA algorithm; (**b**) APF algorithm.

For local path planning performance, we compared the DWA algorithm with the Artificial Potential Field (APF) algorithm. The APF algorithm is a prevalent choice in mobile robotics as it represents a canonical reactive navigation method, providing a meaningful baseline to evaluate planning performance. The simulation results of the two algorithms running separately are illustrated in Figure 8. In DWA algorithm (Figure 8a), the trajectory lengths are 15.32 m, and the path is smoother than that of APF algorithm (Figure 8b). This smoothness suggests that the robot displays consistent changes in angular velocity during movement, resulting in a stable motion posture and reduced jitter during operation. Such stability is advantageous for the robot’s capability in orchard image information collection.

**Figure 9:**
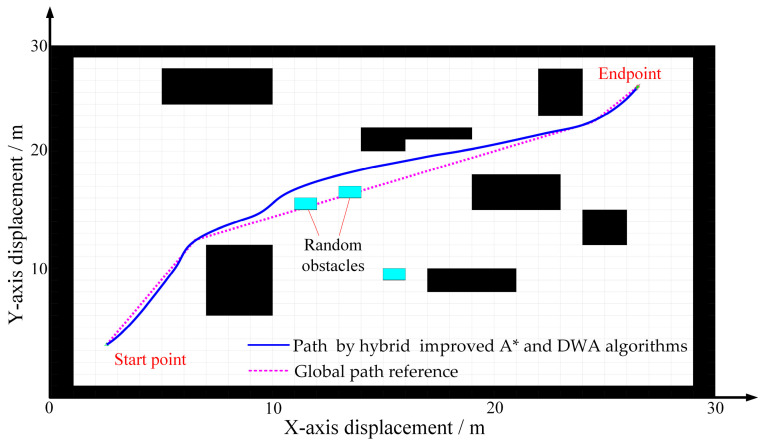
Path planning results based on the combination of A* algorithm and DWA.

Path planning result based on the combination of improved A* algorithm and DWA is shown in Figure 9. The red dashed line represents the global path reference, while the blue solid line depicts the path generated by the combined improved A* and DWA algorithms. The robot successfully navigates around randomly placed temporary obstacles, thereby meeting the path planning and obstacle avoidance criteria outlined in this work.

#### 2.5.2. Calibration of Sensors and Navigation Implementation Steps

The calibration of the LiDAR and IMU sensors was performed to ensure accurate data fusion within the ROS framework. The extrinsic calibration—determining the precise 3D transformation (rotation and translation) between the LiDAR and IMU coordinate frames—was conducted using a standard target-based method. Specifically, the robot was moved in a series of motions in a calibration environment with distinctive geometric features. By collecting synchronized LiDAR point clouds and IMU data during these motions, the transformation parameters were optimized to maximize the consistency between the IMU’s motion estimates and the corresponding point cloud registrations. This process was facilitated by established ROS calibration tools, resulting in a fixed transformation matrix that is accurately published via the TF system.

Steps to initiate the robot navigation function:

1. Launch the chassis driver (*robuster_base.launch*), followed by the mapping program (*mapping.launch*) to begin recording data about the surrounding environment. Through this process, the Rviz tool will visualize the captured information in real time.

2. In remote control mode, manually navigate the robot through the environment to collect point cloud data. After sufficient data has been gathered, execute the map conversion program to save the generated map.

3. Use GNU Image Manipulation Program (GIMP) software to refine the saved map by delineating the operational boundary and removing noise. Export the cleaned map in .*pgm* format.

4. Launch the navigation program (*navigation.launch*) and run Rviz. Load the refined map saved in the step 3. In the Rviz interface, click 2*D Pose Estimate* to set the initial position and orientation of the robot. The system will automatically align itself with the map using environmental data.

5. Click 2*D Nav Goal* to specify a target point on the map. After disabling remote control mode, the robot will navigate toward the selected target at a speed of 0.8 m/s.

All navigation and obstacle avoidance tests were conducted under consistent environmental conditions, specifically clear weather, dry ground, and daytime lighting.

#### 2.5.3. Navigation Path Test

(1) Stopping Test at a Specific Position

**Figure 10:**
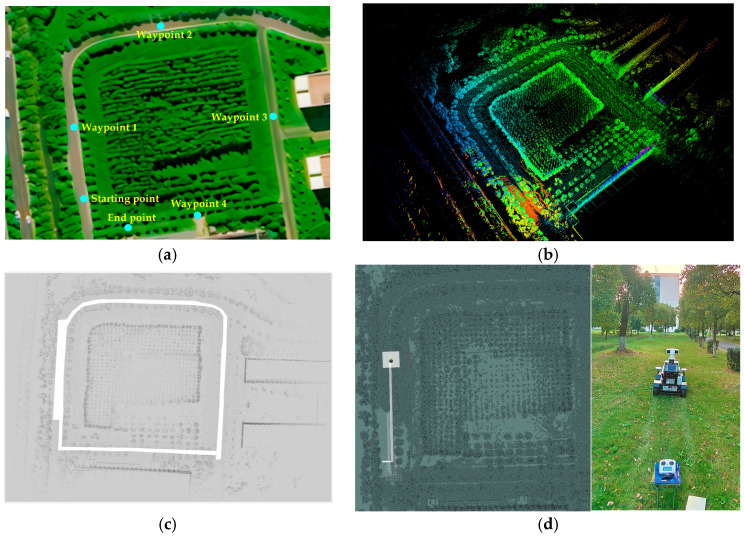
Robot testing scenario: (**a**) waypoint markers; (**b**) navigation mapping of robot operations; (**c**) map refinement; and (**d**) navigation operation.

During robotic inspection operations, it is essential to collect multi-angle information and ensure the stability of image data. To achieve this, the robot must perform turns and pause at designated locations. This study conducted simulated tests to address such operational requirements. The experimental site was situated on the south side of the Lixing Building at Yangzhou University’s Yangzijin Campus. The robot’s stopping positions at the waypoints are depicted in Figure 10a. Four waypoints were established, with the robot pausing for five seconds at each one.

Following the aforementioned steps, the robot initiated map construction based on LiDAR data (Figure 10b). Path boundaries were delineated during the map refinement phase, as illustrated in Figure 10c. Subsequently, the waypoint locations were marked using the following method: the robot was remotely controlled to each waypoint, and the corresponding location was recorded via a node program. After this, the robot returned to the starting point, the navigation endpoint was set, and the navigation process was initiated, as illustrated 10d. Concurrently, data packets were recorded to support subsequent data analysis. The experiment was conducted with three repetitions following the identical protocol.

(2) Obstacle avoidance test

Obstacle avoidance capability is a key indicator of robot performance. After completing the navigation performance tests, this study evaluates the robot’s ability to avoid obstacles. The successful execution of obstacle avoidance depends heavily on the configuration of the costmap. Specifically, within the costmap’s general plugin, the parameter *inflation_radius* defines the inflation radius of obstacles, representing the minimum safe distance the robot must maintain from surrounding obstacles. This study aims to determine the optimal inflation radius to enhance the robot’s obstacle avoidance performance.

**Figure 11:**
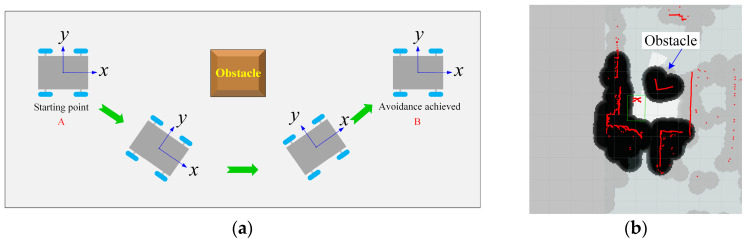
Obstacle avoidance: (**a**) schematic diagram of obstacle avoidance and (**b**) test scene.

The specific steps of tests are as follows:

1. Position the robot in the environment shown in Figure 11a, ensuring that the locations of the obstacles and the robot’s starting point remain consistent throughout the experimental process. The minimum and maximum obstacle heights were set to 0.2 m and 2 m, respectively. The minimum height filters out insignificant ground irregularities while still detecting obstacles high enough to impact the robot’s chassis. Considering the robot’s total height of 1.5 m, the maximum height of obstacle is configured to detect low-hanging branches and other overhead obstacles that could collide with the robot’s superstructure (e.g., the elevating pole and cargo platform), thereby ensuring comprehensive navigational safety in a complex orchard canopy.

2. Follow the navigation function activation steps in Section 2.3 to begin the operation. The robot’s obstacle avoidance effect in Rviz is shown in Figure 11b.

3. Set the inflation radii in the costmap general plugin to 1.0 m, 0.8 m, 0.6 m, 0.4 m, and 0.3 m, respectively, and conduct obstacle avoidance tests for each setting. It is important to restart the upper computer after each adjustment to ensure the updated parameters are correctly loaded.

4. For each inflation radius, perform three trials. In each trial, record whether the robot makes contacts with an obstacle. Document the nearest distance between the robot and the obstacle. Subsequently, calculate the average distance recorded across the trials. All obstacle avoidance tests were conducted with three repetitions for each experimental configuration.

## 3. Results

### 3.1. Stationary Test at Designated Locations

**Figure 12:**
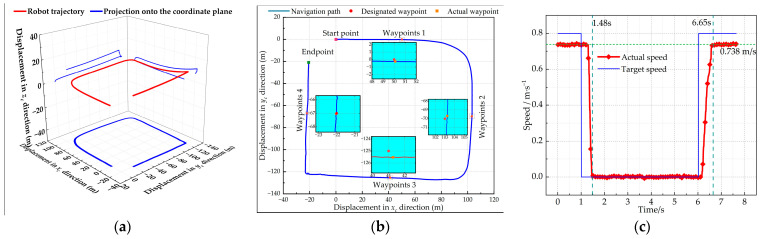
Robot position variation: (**a**) 3D trajectory of the robot; (**b**) comparison of designated waypoints and actual waypoint positions; (**c**) speed variation before and after waypoint 1.

The trajectory of the robot’s movement is illustrated in Figure 12a. Due to minor surface fluctuations, the trajectory shows some variation along the *z_c_* direction. A comparison between the designated waypoints and the actual stopping positions is presented in Figure 10b. Correlating the data in Figure 12b with Table 1 reveals that the robot is able to stop in the vicinity of the four designated waypoints. However, some deviations were observed. Among these waypoints, the maximum lateral error was 0.273 m, with an average lateral error of 0.163 m. The maximum longitudinal error was 0.526 m, with an average of 0.282 m. These deviations are primarily attributed to the robot’s necessity to decelerate and come to a complete at each waypoint, which negatively impacts its stopping precision. Despite these deviations, the robot’s stopping position meets operational requirements, as long as the actual stopping position remains within one meter of the specified waypoint. Additionally, the average orientation error was 2.9°. For inspection robots utilizing wide-angle or panoramic cameras, an average orientation error of 2.9° does not introduce significant degradation in imaging performance. The variations in positional errors are attributed to the unstructured outdoor testing environment, such as minor ground irregularities. The orientation error demonstrates consistency with a small standard deviation, indicating stable and reliable heading control at waypoints. Nevertheless, the achieved average accuracy meets the requirements for robotic inspection tasks.

The robot spends the most time at waypoint 1, with its speed variation curve illustrated in Figure 12c. The figure indicates that while cruising at a target speed of 0.8 m/s, the robot achieves an actual average speed of 0.74 m/s, reflecting a slight deviation from the target due to random ground disturbances. The actual stopping time at the waypoint remains under 5 s, and the robot’s stay time during inspections does not exceed 10 s, thereby meeting the requirements for remaining at specified points for inspection. Upon reaching the waypoint, the stopping time is recorded at 0.48 s, while the time taken to accelerate to the target speed after leaving is 0.65 s. The average braking time and startup time of the robot at the four waypoint locations are 0.46 s and 0.64 s, respectively, demonstrating a satisfactory speed response.

### 3.2. Obstacle Avoidance Test

The results of the robot’s obstacle avoidance tests are summarized in Table 2. The effectiveness of obstacle avoidance is evaluated based on three criteria: whether the robot becomes disoriented during navigation, whether it makes contacts with obstacles, and the closest distance to obstacles. A larger expansion radius results in a greater boundary range around obstacles, which theoretically decreases the likelihood of collisions. However, when the inflation radius is set to 1.0 m, the robot becomes disoriented and fails to reach the target point. This failure is primarily due to the close spacing of obstacles, which restricts navigable paths and confuses the robot’s trajectory planning. Therefore, it is advisable to reduce the expansion radius. The robot avoids obstacles when the inflation radius is set to 0.8 m, 0.6 m, and 0.4 m.

However, when comparing the three trajectories, the path with an inflation radius of 0.8 m results in an excessively large turning radius. Similarly, at an expansion radius of 0.6 m, the robot exhibits overly sharp turns during the initial stages of obstacle avoidance. In contrast, the path corresponding to an inflation radius of 0.4 m demonstrates the most gradual and efficient adjustments. At an expansion radius of 0.3 m, the robot collides with obstacles. Considering both the robot’s dimensions and the experimental results presented in this paper, an expansion radius of 0.4 m is determined to be optimal. This setting allows the robot to reach the target point while maintaining an adequate safe distance from surrounding obstacles.

## 4. Discussion

This study presents a comprehensive investigation into the SLAM capabilities of a mobile inspection robot using LiDAR-based perception. A key innovation lies in the development of a novel multi-sensor platform that integrates LiDAR and an IMU, enabling robust environmental perception and precise localization. Additionally, a Pan-Tilt-Zoom camera is incorporated to support adaptive visual inspections, thereby enhancing situational awareness in dynamic environments. To ensure accurate motion representation, a detailed kinematic model with coordinate transformations was established and implemented using the URDF for seamless visualization in ROS. For autonomous navigation, an optimized path planning strategy was employed, combining the global A* algorithm with the DWA for real-time, collision-free trajectory optimization. This integrated framework not only improves SLAM performance but also demonstrates significant advancements in robotic autonomy and adaptability in complex, real-world scenarios.

To the best of our knowledge, there is a relative lack of research exploring the synergy between ROS parameters and overall robot performance. This paper provides a comprehensive quantitative analysis of the configuration of costmap plugins, including general plugins, global costmap plugins, and local costmap plugins, along with the path planning plugin and the launch configurations for navigation nodes. By integrating the robot’s URDF model, developed from a physical 3D model, with the transformation framework of the robot’s coordinates, path information can be efficiently converted into motion control commands. This integration established a cohesive link between ROS parameters and robot parameters, ultimately improving the effectiveness of autonomous navigation.

The existing literature includes numerous studies on SLAM-based navigation path planning for mobile robots through the integration of A* and DWA algorithms. Representative examples include: (1) Path planning for mobile robots based on improved A* and fuzzy DWA algorithms [27]; (2) research on robot path planning by integrating state-based decision-making A* algorithm and inertial DWA [28]; and (3) hybrid path planning based on safe A* algorithm and adaptive window approach for mobile robot in large-scale dynamic environments [29]. However, these studies primarily focus on structural enhancements of the algorithms themselves, with relatively limited emphasis on the synergistic integration between algorithmic frameworks and robotic navigation environments. This marks a key distinction from the present study, which emphasizes environment-algorithm co-adaptation in complex operational contexts.

The experimental results of this study also demonstrate the robot performed well in temporarily stopping at designated waypoints, reflecting effective coordination between the path planning and control systems. This capability is essential for achieving precise operations in inspection tasks [30].

The obstacle avoidance experiments conducted with varying inflation radii indicated that an inflation radius of 0.4 m yielded the most effective results. This finding provides a reference for practical robot applications, enabling future users to adjust this parameter flexibly based on environmental conditions to optimize operational efficiency and safety. Additionally, the result validates the effectiveness of DWA in real-time obstacle avoidance, highlighting the robot’s potential for deployment in complex environments [31].

Finally, despite the promising results, in geometrically degraded environments like homogeneous orchards, LiDAR point clouds lack distinct features, challenging scan-matching algorithms and increasing pose estimation uncertainty. The kinematic model used for motion control and odometry calculation typically assumes ideal wheel-ground contact. Localization drift in feature-sparse areas and the influence of ground irregularities on motion control may contribute to positional errors. Recent studies demonstrate enhanced robustness by combining A* with adaptive fuzzy controllers in ROS2 to mitigate motion disturbances [32], and improved navigation in dynamics via deep reinforcement learning [33]. These approaches present a compelling direction for evolving our system beyond classical planning, through the integration of adaptive control and learned policies.

## 5. Conclusions

This study developed and validated a LiDAR-based SLAM system for a mobile inspection robot, integrating tightly coupled multi-sensor fusion to enhance environmental perception and autonomous navigation. The proposed robotic platform, combining LiDAR and IMU data, demonstrated robust localization and mapping capabilities. A precise kinematic model was developed using the URDF framework, enabling efficient coordinate transformations and real-time visualization in ROS. A path planning strategy, combining an improved A* algorithm with DWA, was introduced to enhance navigation efficiency and obstacle avoidance. Field tests evaluated the robot’s path navigation accuracy, and obstacle avoidance capabilities. Under temporary parking conditions at designated waypoints, the robot achieved a maximum lateral deviation of 0.273 m and a maximum longitudinal deviation of 0.526 m, with a parking time of 0.48 s and an acceleration duration of 0.65 s. Velocity response characteristics met precision requirements for waypoint parking operations. Obstacle avoidance experiments identified an optional inflation radius of 0.4 m, providing a baseline configuration while allowing for scenario-specific fine-tuning in inspection tasks. These findings confirm that the proposed hybrid path planning method can effectively generate collision-free navigation paths for mobile robots. The developed algorithm demonstrates strong potential for deployment in complex environments, offering a robust and scalable framework for autonomous navigation system design. These contributions advance the field of autonomous robotics by providing a scalable framework for multi-sensor SLAM and intelligent navigation, with potential applications in industrial inspection, agricultural monitoring, and other mission-critical operations.

This study has several limitations. The test scenarios were relatively limited. Sensitivity to sensor calibration and environmental dynamics have not been adequately considered. Future work will focus on adaptive neural frameworks and large-scale field validation to enhance robustness and scalability.

Future work will focus on the practical application scenarios of robots, performance optimization under dynamic conditions, and the integration of adaptive neural frameworks to further enhance autonomous learning capabilities.

## Figures and Tables

**Table 1:** Deviations in waypoint positions.

Types of Errors	Waypoint 1	Waypoint 2	Waypoint 3	Waypoint 4	Average Value	Standard Deviation
lateral error/m	0.097	0.241	0.273	0.040	0.163	0.112
Longitudinal error/m	0.253	0.310	0.526	0.040	0.282	0.200
Orientation Error (°)	2.1	3.5	4.2	1.8	2.9	1.140

**Table 2:** Robot obstacle avoidance test record.

Expansion Radius Value/m	Fall into Trajectory Confusion	Collision with Obstacles	Closest Distance to Obstacles/m
1.0	Yes	No	1.284
0.8	No	No	0.826
0.6	No	No	0.643
0.4	No	No	0.375
0.3	No	Yes	0

## Data Availability

The original contributions presented in this study are included in the article. Further inquiries can be directed to the corresponding author.
